# The association of clinical and patient factors with chemotherapy-induced peripheral neuropathy (CIPN) in colorectal cancer: secondary analysis of the SCOT trial

**DOI:** 10.1016/j.esmoop.2023.102063

**Published:** 2023-11-17

**Authors:** A. Lemanska, A. Harkin, T. Iveson, C. Kelly, M. Saunders, S. Faithfull

**Affiliations:** 1 Faculty of Health and Medical Sciences, University of Surrey, Guildford, UK; 2 Cancer Research UK Glasgow Clinical Trials Unit, Glasgow, UK; 3 Department of Medical Oncology, University of Southampton, Southampton, UK; 4 Christie Hospital, Manchester, UK; 5 School of Medicine, Trinity College, Dublin, Ireland

**Keywords:** oxaliplatin, neuropathy, chemotherapy-induced peripheral neuropathy, CIPN, colorectal cancer

## Abstract

**Background:**

Chemotherapy-induced peripheral neuropathy (CIPN) is a common adverse effect of oxaliplatin. CIPN can impair long-term quality of life and limit the dose of chemotherapy. We investigated the association of CIPN over time with age, sex, body mass index, baseline neuropathy, and chemotherapy regimen in people treated with adjuvant oxaliplatin-containing chemotherapy for colorectal cancer.

**Patients and methods:**

We carried out secondary analysis of data from the SCOT randomised controlled trial. SCOT compared 3 months to 6 months of oxaliplatin-containing adjuvant chemotherapy in 6088 people with colorectal cancer recruited between March 2008 and November 2013. Two different chemotherapy regimens were used: capecitabine with oxaliplatin (CAPOX) or fluorouracil with oxaliplatin (FOLFOX). CIPN was recorded with the Functional Assessment of Cancer Therapy/Gynaecologic Oncology Group-Neurotoxicity 4 tool in 2871 participants from baseline (randomisation) for up to 8 years. Longitudinal trends in CIPN [averages with 95% confidence intervals (CIs)] were plotted stratified by the investigated factors. Analysis of covariance (ANCOVA) was used to analyse the association of factors with CIPN adjusting for the SCOT randomisation arm and oxaliplatin dose. *P* < 0.01 was adopted as cut-off for statistical significance to account for multiple testing.

**Results:**

Patients receiving CAPOX had lower CIPN scores than those receiving FOLFOX. Chemotherapy regimen was associated with CIPN from 6 months (*P* < 0.001) to 2 years (*P* = 0.001). The adjusted ANCOVA coefficient for CAPOX at 6 months was −1.6 (95% CIs −2.2 to −0.9) and at 2 years it was −1.6 (95% CIs −2.5 to −0.7). People with baseline neuropathy scores ≥1 experienced higher CIPN than people with baseline neuropathy scores of 0 (*P* < 0.01 for all timepoints apart from 18 months). Age, sex, and body mass index did not link with CIPN.

**Conclusions:**

A neuropathy assessment before treatment with oxaliplatin can help identify people with an increased risk of CIPN. More research is needed to understand the CIPN-inducing effect of different chemotherapy regimens.

## Highlights

• Patients with neuropathy at baseline experienced higher acute and long-term CIPN scores than those without.
• CAPOX regimen was beneficial in terms of CIPN. Patients receiving CAPOX had lower CIPN scores than those receiving FOLFOX.
• Age, sex, and body mass index were not associated with CIPN.
• A simple neuropathy assessment before treatment with oxaliplatin could help identify people with an increased risk of CIPN.

## Introduction

Chemotherapy-induced peripheral neuropathy (CIPN) is a common adverse effect of platinum-based derivatives such as oxaliplatin. CIPN can limit the ability to tolerate treatment and can negatively affect patients’ long-term quality of life and physical functioning.^1^, ^2^, ^3^ Oxaliplatin is used as part of adjuvant treatment for stage II and III colorectal cancer, but it is associated with long-term neurotoxicity which is dose dependent,^4^ and is the most common dose-limiting side-effect.^5^ Long-term CIPN can cause substantial disability. There is a need to identify those most at risk of developing CIPN to mitigate symptoms through treatment alterations balanced with maintaining treatment efficacy.^5^ At present there is no evidence-based interventions that prevent or reverse CIPN. Current clinical practice guidelines by the European Society for Medical Oncology (ESMO, 2020) recommend a neurological assessment before treatment to identify subclinical neuropathy as well as a specialist referral for high-risk patients.^6^ In addition, the American Society of Clinical Oncology guidelines recommend that clinicians assess the appropriateness of dose reductions, dose delays, and substitutions in patients who develop severe acute CIPN.^7^

Chemotherapy-induced nerve damage occurs in two phases, acute and long-term, with differences in timing and symptom trajectory.^8^ Acute symptoms have a rapid onset, are transient, and characterised by cold-induced distal sensory symptoms such as paraesthesia and dysaesthesia. Long-term CIPN occurs months to years after treatment and is characterised by distal sensory symptoms with ataxia, leading to functional impairment.^9^ However, the exact mechanisms of CIPN are unclear.^10^^,^^11^ Clinically, CIPN symptoms manifest especially in the hands and feet, with sensory loss, numbness, tingling, pain, thermal sensitivity, and motor symptoms impacting co-ordination,^12^ balance, and increasing falls.^13^ A recent meta-analysis in people treated for colorectal cancer found the prevalence of CIPN to be 58% at 6 months, 45% at 12 months, 32% at 24 months, and 24% at 36 months after treatment.^14^ In addition, oxaliplatin exhibits a coasting phenomenon, in which CIPN symptoms continue to worsen for ∼3 months after treatment.^15^ Overall recovery from CIPN is also inconsistently reported, with very few studies evaluating longitudinal data and repeated long-term measures.^14^

Factors that predispose CIPN, that have been reported previously, include age >65 years,^16^ low haemoglobin levels,^17^ higher body mass index (BMI),^18^^,^^19^ diabetes,^20^^,^^21^ and history of neuropathy.^22^ However, studies of patient-related CIPN risk factors often show conflicting results. This is potentially due to the heterogeneous population cohorts, small sample sizes, different neurotoxic drugs, and differing methods and timelines of assessment.^14^^,^^23^ A total dose of oxaliplatin is a treatment-related factor that has been consistently associated with greater neurotoxicity.^24^ Recent clinical trials demonstrated that 3 months compared to 6 months of adjuvant oxaliplatin-containing chemotherapy had similar efficacy in terms of overall survival but people receiving fewer cycles (lower cumulative dose) had lower CIPN scores.^25^, ^26^, ^27^, ^28^, ^29^, ^30^ These trials, including the SCOT trial from which data are used in this study, influenced current guidelines and established the 3-month treatment as standard practice.^31^ However, despite this and the associated dose reductions, a proportion of patients will still experience CIPN.^32^ There is a paucity of long-term CIPN data and studies investigating factors that should be taken into account when making treatment decisions.

SCOT was an international, multicentre (244 centres), randomised clinical trial that tested non-inferiority of 3 months versus 6 months of adjuvant oxaliplatin-containing chemotherapy in 6088 people with high-risk stage II and III colorectal cancer recruited between March 2008 and November 2013.^27^ It investigated the effect of chemotherapy duration (randomisation arm) on disease-free survival, quality of life, and CIPN.^27^ In this secondary analysis of SCOT data, we investigated the relationship of patient factors such as age, sex, BMI, and baseline peripheral neuropathy, as well as a clinical factor, namely chemotherapy regimen, with CIPN over time. Our objectives were to demonstrate longitudinal trends in CIPN stratifying by SCOT randomisation arm and patient and clinical factors, and to statistically analyse the significance of these factors on CIPN.

## Patients and methods

### Study design, data source

We carried out secondary analysis of data from the SCOT clinical trial.^27^ We investigated patient factors (age, sex, BMI, baseline peripheral neuropathy) and a clinical factor (chemotherapy regimen) and their relationship with the acute and long-term CIPN.

### Participants

The study sample included 2871 participants who provided CIPN data (of the total 6088 SCOT cohort). As per the SCOT’s design, the CIPN sub-study of SCOT did not require the same number of participants as the main evaluation and the recruitment target was 1800 participants. CIPN data were collected from all recruited participants until the required number was reached. The discontinuation of data collection was endorsed by SCOT’s independent data-monitoring committee and SCOT’s steering committee, based on an interim data analysis and accounting for missing data. Further details of the methods and outcomes for the SCOT trial are reported in the study by Iveson et al. 2018.^27^

### Factors of interest

We accessed patient factors including age at baseline, sex, height and weight at baseline (which we used to calculate BMI), baseline neuropathy, and chemotherapy regimen. Two chemotherapy regimens were allowed in SCOT—fluorouracil with oxaliplatin (FOLFOX) or capecitabine with oxaliplatin (CAPOX). The regimen was decided by clinicians before randomisation. The dose of oxaliplatin in FOLFOX was 85 mg/m^2^ every 2 weeks and in CAPOX it was 130 mg/m^2^ every 3 weeks for the duration of treatment which was randomised. The two SCOT randomisation arms were the 3-month arm (six cycles of FOLFOX delivered every 2 weeks or four cycles of CAPOX delivered every 3 weeks) and the 6-month arm (12 cycles FOLFOX delivered every 2 weeks or 8 cycles of CAPOX delivered every 3 weeks). The effect of the treatment duration (randomisation arm) on CIPN was assessed in the main publication from the SCOT trial published by Iveson et al. in 2018,^27^ and was not the focus of this study. The planned total oxaliplatin dose in the 3-month arm was 510 mg/m^2^ or 520 mg/m^2^ in the FOLFOX and CAPOX regimens, respectively, and in the 6-month arm it was 1020 mg/m^2^ and 1040 mg/m^2^, respectively. No dose reductions of oxaliplatin were allowed at baseline.

### Outome measures

Peripheral neuropathy was assessed with a validated patient-reported outcome measure, the Functional Assessment of Cancer Therapy/Gynaecologic Oncology Group-Neurotoxicity 4 (FACT/GOG-NTX-4) tool.^33^ FACT/GOG-NTX-4 is a reduced four-item version of the FACT neurotoxicity subscale and can be obtained from www.facit.org/measures/FACT-GOG-NTX-4. It assesses numbness or tingling in the hands and feet and a feeling of discomfort in the hands and feet, and provides a single aggregate score for peripheral neuropathy. Peripheral neuropathy was assessed in SCOT at randomisation (baseline), then monthly for up to 6 months, then at 9, 12, 18, and 24 months, and then annually for up to 8 years.

### Statistical analysis

Descriptive statistics including means with standard deviations, medians with interquartile ranges, and counts with percentages were used to summarise patient characteristics and clinical data. Data were presented for the total study sample and separately for (i) two groups according to the SCOT randomisation arm, and (ii) two groups according to the chemotherapy regimen. The statistical significance of the difference between the groups was assessed with the *t*-test for means, Wilcoxon test for medians, and chi-square test for counts data.

Means with 95% confidence intervals (CIs) were used to visualise the longitudinal trends in peripheral neuropathy from baseline for up to 6 years stratified by the SCOT randomisation arm and the study factors including age (≤65 versus>65 years), sex (male versus female), BMI (≤25 versus >25), baseline peripheral neuropathy score (0 versus ≥1), and chemotherapy regimen (FOLFOX versus CAPOX). SCOT assessed peripheral neuropathy for up to 8 years. However, due to a high proportion of missing data we present these data for up to 6 years.

Analysis of covariance (ANCOVA) was used to estimate the difference in CIPN scores over time (from 3 months to 24 months) between the groups according to age, sex, BMI, baseline peripheral neuropathy, and chemotherapy regimen. The statistical significance of the difference was estimated using 95% CIs. ANCOVA is a regression-based method appropriate for trial data. It allows multivariable analysis and adjusting for the oxaliplatin total dose received and the randomisation arm which was included as a covariate (the effect of treatment duration on CIPN was investigated elsewhere).^27^ An ANCOVA coefficient represents the magnitude of the effect associated with a factor. The 95% CIs for the coefficient that do not span 0 represent statistically significant results at *P* < 0.05. We presented 95% CIs to aid comparisons with other published studies. However, due to multiple statistical tests, we used *P* < 0.01 as a cut-off level for deciding statistical significance. Analyses were undertaken according to the intention-to-treat principle and included all randomised SCOT participants who participated in the CIPN sub-study. The proportion of missing data is described but missing data were not imputed (we used complete case analysis). Statistical analyses were carried out in R version 4.0.2.

### Ethical considerations

The study protocol was approved by the SCOT steering group. Ethical approval (FHMS 20-21 142 EGA) was granted by the University of Surrey Ethics Committee on 28 May 2021.

## Results

Baseline patient characteristics and clinical data are summarised in Table 1. This was for the total study sample, as well as stratified by the SCOT randomisation arm and according to the chemotherapy regimen. The average age of participants at randomisation was 63 years (±9 standard deviation). Most of the participants (94%, *n* = 2694) were of White ethnicity and 39% (*n* = 1110) were females. Colon cancer was more prevalent than cancer of the rectum (81%, *n* = 2324). Patient characteristics and clinical factors were balanced across the groups according to randomisation arms and chemotherapy regimens. The only significant difference was recorded for ethnicity between the chemotherapy regimen groups (*P* = 0.001), and it was due to a larger proportion of missing data in the FOLFOX group.

**Table 1:** Baseline characteristics of the study sample

Characteristic	Total sample	By SCOT’s randomisation arm			By chemotherapy regimen		
	*N* = 2871	3 months arm, *n* = 1445	6 months arm, *n* = 1426	*P* value	FOLFOX, *n* = 925	CAPOX, *n* = 1946	*P* value
Age (years)							
Mean (SD)	63 (9)	63 (9)	64 (10)	0.645	64 (9)	63 (10)	0.334
Median (IQR)	65 (59-70)	64 (58-70)	65 (59-70)	0.416	65 (59-70)	64 (58-70)	0.406
BMI (kg/m^3^), *n* (%)				0.692			0.884
≤25	1047 (37%)	521 (37%)	526 (37%)		340 (37%)	707 (37%)	
>25	1779 (63%)	899 (63%)	880 (63%)		573 (63%)	1206 (63%)	
Missing, *n* (%)	45 (2%)	25 (2%)	20 (1%)		12 (1%)	33 (2%)	
Sex, *n* (%)				0.799			0.846
Female	1110 (39%)	562 (39%)	548 (38%)		360 (39%)	750 (39%)	
Male	1761 (61%)	883 (61%)	878 (62%)		565 (61%)	1196 (61%)	
Ethnicity, *n* (%)				0.064			0.001
White	2694 (94%)	1344 (93%)	1350 (95%)		841 (91%)	1853 (95%)	
Other	118 (4%)	68 (5%)	50 (3%)		42 (4%)	76 (4%)	
Missing, *n* (%)	59 (2%)	33 (2%)	26 (2%)		42 (4%)	17 (1%)	
Performance status, *n* (%)				0.547			0.616
0	1958 (68%)	993 (69%)	965 (68%)		625 (68%)	1333 (68%)	
1	913 (32%)	452 (31%)	461 (32%)		300 (32%)	613 (32%)	
Disease site, *n* (%)				0.948			0.628
Colon	2324 (81%)	1169 (81%)	1155 (81%)		744 (80%)	1580 (81%)	
Rectum	547 (19%)	276 (19%)	271 (19%)		181 (20%)	366 (19%)	
Chemotherapy regimen, *n* (%)				0.908			
FOLFOX	925 (32%)	467 (32%)	458 (32%)				
CAPOX	1946 (68%)	978 (68%)	968 (68%)				
Oxaliplatin total dose (mg/m^2^)							
3-month arm, mean (SD)					459 (108)	443 (120)	0.010
6-month arm, mean (SD)					693 (250)	670 (286)	0.125
Baseline neuropathy score, *n* (%)				0.381			0.447
0	1407 (49%)	694 (48%)	713 (50%)		457 (49%)	950 (49%)	
≥1	317 (11%)	165 (11%)	152 (11%)		110 (12%)	207 (11%)	
Missing, *n* (%)	1147 (40%)	586 (41%)	561 (39%)		358 (39%)	789 (41%)	

Total study sample, by SCOT randomisation arm and by chemotherapy regimen. The statistical significance of the difference between the groups was assessed with the two-sample *t*-test for means, Wilcoxon test for medians, and chi-square test for counts data. Number of missing data (if present) is indicated as *n* and %; proportions are calculated for available data.

BMI, body mass index; CAPOX, capecitabine with oxaliplatin; FOLFOX, fluorouracil with oxaliplatin; IQR, interquartile range; SD, standard deviation.

### Longitudinal profiles of peripheral neuropathy

**Figure 1:**
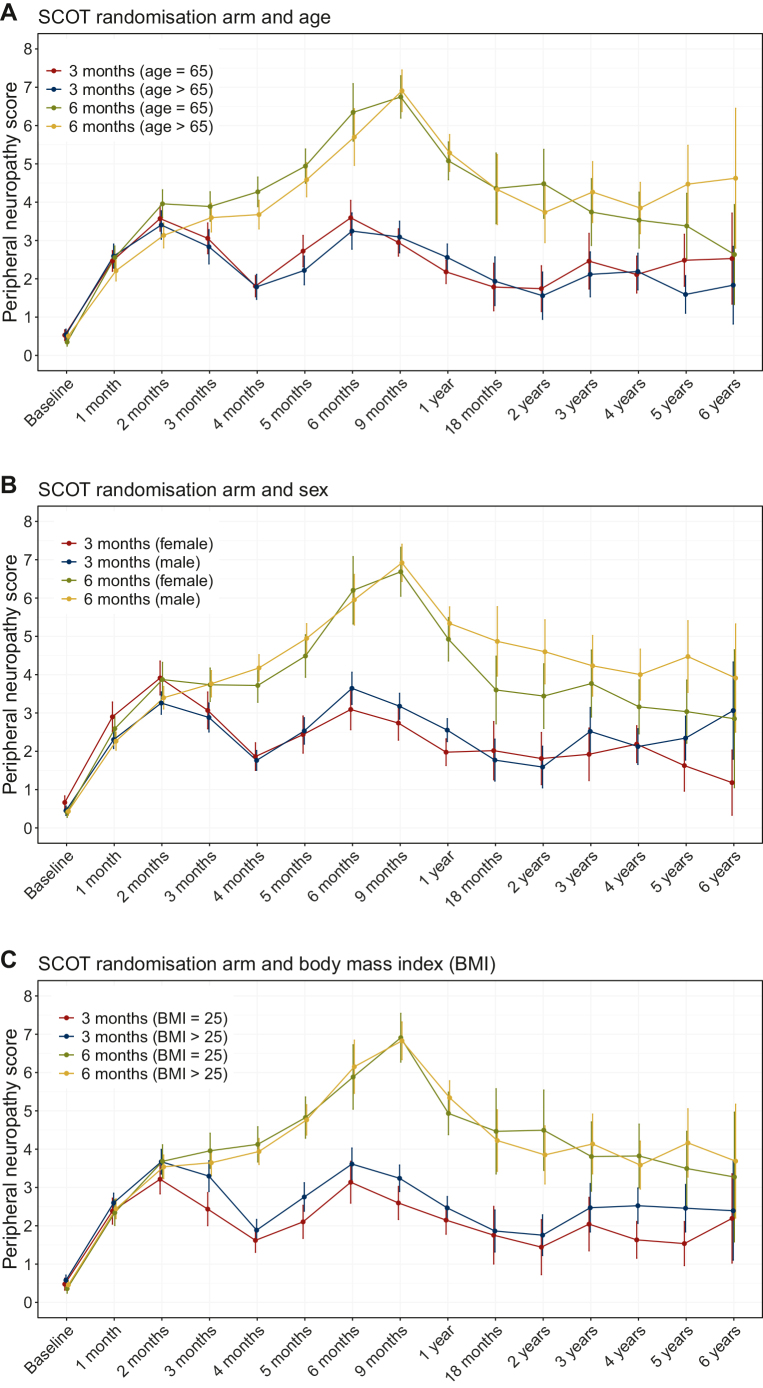

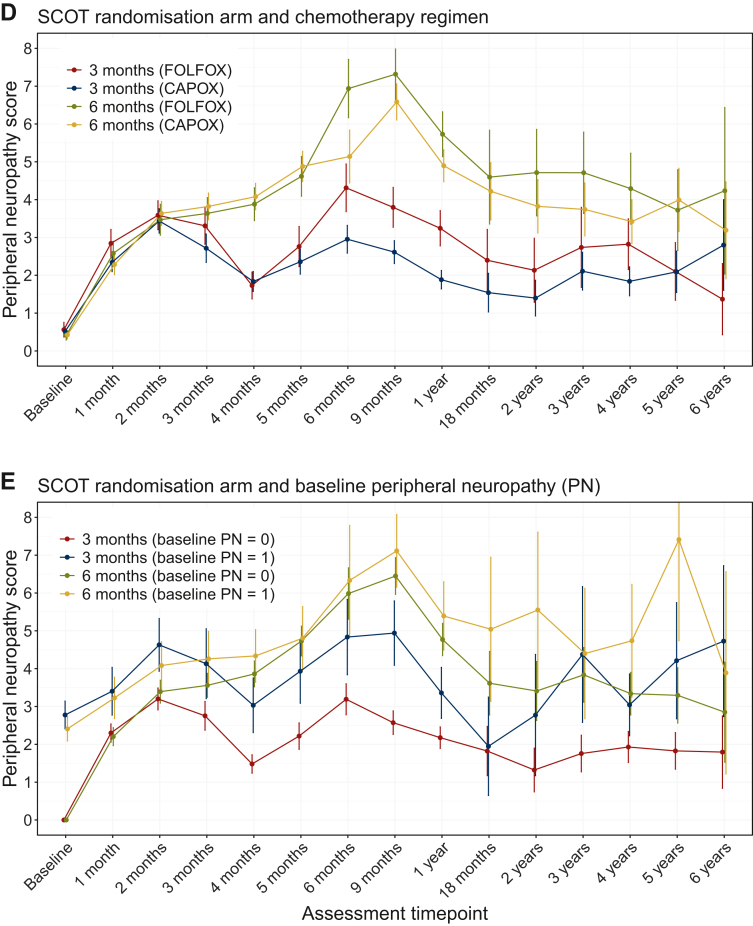
Longitudinal profiles of chemotherapy-induced peripheral neuropathy (CIPN). Means with 95% confidence intervals by the SCOT randomisation arm and a stratifying factor: (A) age; (B) sex; (C) body mass index (BMI); (D) chemotherapy regimen; and (E) baseline peripheral neuropathy.

Longitudinal profiles of peripheral neuropathy are presented in Figure 1. The differences in peripheral neuropathy trends according to the SCOT randomisation arm have been described elsewhere.^27^ In this study we focus on patient factors and chemotherapy regimen.

There were no statistically significant differences in peripheral neuropathy scores according to age, sex, or BMI (Figure 1A-C). However, statistically significant differences were observed between the groups according to chemotherapy regimen and baseline neuropathy (Figure 1D and E). Figure 1D shows differing trajectories of CIPN between the FOLFOX and CAPOX regimens. From 6 months onwards, patients who received FOLFOX experienced higher CIPN scores than those who received CAPOX.

In addition, baseline peripheral neuropathy affected peripheral neuropathy scores during and after treatment (Figure 1E). People with baseline peripheral neuropathy scores of ≥1 had higher CIPN than people with a baseline peripheral neuropathy score of 0, and this was especially prominent in the 3-month group. When stratified by baseline neuropathy, after 1 year, the trajectory became distorted and was associated with wide CIs due to large proportions of missing data.

### Association of patient and clinical factors with CIPN

ANCOVA results (Table 2) demonstrate the association of chemotherapy regimen and baseline peripheral neuropathy with CIPN. CAPOX was associated with lower CIPN scores than FOLFOX. The statistically significant differences in CIPN due to the regimen started at 6 months (the adjusted ANCOVA coefficient for CAPOX was −1.6, 95% CIs −2.3 to −0.9) and continued for up 24 months (−1.5, 95% CIs −2.4 to −0.6) when we ceased the ANCOVA analysis.

**Table 2:** ANCOVA for the effect of age, sex, BMI, chemotherapy regimen (CAPOX versus FOLFOX), baseline PN, and oxaliplatin dose on acute and long-term CIPN from 3 to 24 months

	Univariable models			Multivariable models		
	Coefficient	95% CI	*P* value	Coefficient	95% CI	*P* value
Month 3						
Age (10 years)	−0.1	(−0.3 to 0.1)	0.225	0.1	(−0.2 to 0.3)	0.526
Sex (female)	0.0	(−0.5 to 0.4)	0.821	0.0	(−0.5 to 0.4)	0.839
BMI	0.0	(0.0 to 0.1)	0.183	0.0	(0.0 to 0.1)	0.268
Regimen (CAPOX)	−0.1	(−0.5 to 0.3)	0.559	−0.2	(−0.7 to 0.2)	0.333
Baseline PN (≥1)	1.0	(0.4 to 1.6)	0.001∗	0.9	(0.3 to 1.5)	0.004∗
Oxaliplatin total dose (100 mg/m^2^)	0.0	(−0.1 to 0.1)	0.517	−0.1	(−0.2 to 0.0)	0.166
Month 4						
Age (10 years)	−0.2	(−0.4 to 0.0)	0.087	−0.1	(−0.3 to 0.1)	0.289
Sex (female)	0.2	(−0.2 to 0.6)	0.341	0.1	(−0.3 to 0.6)	0.490
BMI	0.0	(0.0 to 0.0)	0.746	0.0	(0.0 to 0.1)	0.682
Regimen (CAPOX)	0.1	(−0.3 to 0.5)	0.625	−0.1	(−0.5 to 0.3)	0.657
Baseline PN (≥1)	1.0	(0.4 to 1.5)	<0.001∗	1.0	(0.5 to 1.5)	<0.001∗
Oxaliplatin total dose (100 mg/m^2^)	0.3	(0.2 to 0.4)	<0.001∗	0.0	(−0.1 to 0.1)	0.841
Month 5						
Age (10 years)	−0.3	(−0.5 to 0.0)	0.033	−0.2	(−0.4 to 0.1)	0.201
Sex (female)	0.3	(−0.1 to 0.8)	0.173	0.1	(−0.4 to 0.7)	0.600
BMI	0.0	(0.0 to 0.1)	0.225	0.0	(0.0 to 0.1)	0.671
Regimen (CAPOX)	−0.1	(−0.6 to 0.3)	0.535	−0.1	(−0.7 to 0.4)	0.576
Baseline PN (≥1)	0.8	(0.2 to 1.5)	0.008∗	1.0	(0.4 to 1.6)	0.002∗
Oxaliplatin total dose (100 mg/m^2^)	0.4	(0.3 to 0.5)	<0.001∗	0.1	(−0.1 to 0.2)	0.218
Month 6						
Age (10 years)	−0.1	(−0.5 to 0.2)	0.407	−0.1	(−0.5 to 0.3)	0.587
Sex (female)	0.2	(−0.4 to 0.8)	0.522	0.1	(−0.6 to 0.7)	0.828
BMI	0.0	(0.0 to 0.1)	0.627	0.0	(−0.1 to 0.1)	0.980
Regimen (CAPOX)	−1.8	(−2.4 to −1.2)	<0.001∗	−1.6	(−2.3 to −0.9)	<0.000∗
Baseline PN (≥1)	1.3	(0.4 to 2.2)	0.003∗	1.2	(0.4 to 2.0)	0.004∗
Oxaliplatin total dose (100 mg/m^2^)	0.5	(0.4 to 0.7)	<0.001∗	0.2	(0.0 to 0.5)	0.064
Month 9						
Age (10 years)	0.2	(0.0 to 0.5)	0.096	0.2	(−0.1 to 0.5)	0.145
Sex (female)	0.3	(−0.2 to 0.8)	0.296	0.0	(−0.5 to 0.5)	0.929
BMI	0.0	(0.0 to 0.1)	0.390	0.0	(0.0 to 0.1)	0.701
Regimen (CAPOX)	−1.0	(−1.5 to −0.4)	<0.001∗	−0.8	(−1.3 to −0.3)	0.002∗
Baseline PN (≥1)	1.6	(0.9 to 2.3)	<0.001∗	1.5	(0.9 to 2.1)	<0.001∗
Oxaliplatin total dose (100 mg/m^2^)	1.0	(0.9 to 1.1)	<0.001∗	0.8	(0.6 to 0.9)	<0.001∗
Month 12						
Age (10 years)	0.4	(0.2 to 0.6)	0.001∗	0.3	(0.1 to 0.6)	0.009∗
Sex (female)	0.4	(0.0 to 0.9)	0.069	0.3	(−0.2 to 0.8)	0.212
BMI	0.0	(0.0 to 0.1)	0.031	0.0	(0.0 to 0.1)	0.405
Regimen (CAPOX)	−1.1	(−1.6 to −0.6)	<0.001∗	−1.1	(−1.6 to −0.6)	<0.001∗
Baseline PN (≥1)	0.9	(0.3 to 1.5)	0.006	0.8	(0.2 to 1.4)	0.006∗
Oxaliplatin total dose (100 mg/m^2^)	0.7	(0.7 to 0.8)	<0.001∗	0.6	(0.5 to 0.7)	<0.001∗
Month 18						
Age (10 years)	0.2	(−0.2 to 0.7)	0.331	0.2	(−0.2 to 0.7)	0.332
Sex (female)	0.4	(−0.5 to 1.2)	0.389	0.3	(−0.7 to 1.2)	0.559
BMI	0.0	(0.0 to 0.1)	0.381	0.0	(−0.1 to 0.1)	0.722
Regimen (CAPOX)	−0.5	(−1.4 to 0.3)	0.232	−1.1	(−2.1 to −0.2)	0.020
Baseline PN (≥1)	1.0	(−0.3 to 2.2)	0.136	0.7	(−0.4 to 1.9)	0.220
Oxaliplatin total dose (100 mg/m^2^)	0.7	(0.5 to 0.9)	<0.001∗	0.5	(0.2 to 0.7)	<0.001∗
Month 24						
Age (10 years)	0.1	(−0.3 to 0.5)	0.736	0.0	(−0.4 to 0.5)	0.926
Sex (female)	0.3	(−0.5 to 1.1)	0.477	0.4	(−0.6 to 1.3)	0.427
BMI	0.0	(0.0 to 0.1)	0.395	0.0	(−0.1 to 0.1)	0.720
Regimen (CAPOX)	−0.8	(−1.7 to 0.0)	0.045	−1.5	(−2.4 to −0.6)	0.002∗
Baseline PN (≥1)	1.8	(0.6 to 3.0)	0.003∗	1.8	(0.7 to 3.0)	0.001∗
Oxaliplatin total dose (100 mg/m^2^)	0.6	(0.4 to 0.7)	<0.001∗	0.3	(0.1 to 0.5)	0.013

Multivariate models are adjusted for the randomisation arm. The cut-off for statistical significance was set at <0.01 to account for multiple testing.

ANCOVA, analysis of covariance; BMI, body mass index; CAPOX, capecitabine with oxaliplatin; CIPN, chemotherapy-induced peripheral neuropathy; FOLFOX, fluorouracil with oxaliplatin; PN, peripheral neuropathy.

∗ Statistical significance.

In addition, baseline peripheral neuropathy was associated with peripheral neuropathy scores during and after treatment (*P* < 0.01 at all timepoints but 18 months). CIPN was higher for people with baseline peripheral neuropathy (score ≥1) than for people without baseline peripheral neuropathy (score = 0). The adjusted ANCOVA coefficients for the group with the baseline peripheral neuropathy scores ≥1 were 0.9 (95% CI 0.3-1.5) at 3 months, 1.2 (95% CI 0.4-2.0) at 6 months, and 0.8 (95% CI 0.2-1.4) at 12 months.

### Missing data

Ethnicity was missing for 59 (2%) study participants and BMI was missing for 45 (2%). No missing data were reported for other patient and clinical factors. However, severe missing data were reported for peripheral neuropathy. At baseline, peripheral neuropathy scores were missing for 1147 (40%) study participants. This was increasing over time, and was 44%, 46%, 57%, 54%, 56%, 70%, 51%, and 50% at month 1 to month 12. Missing data reached 88% at both 18-month and 24-month peripheral neuropathy assessments. This is represented by wide CIs in the analysis and indicates that the results, especially for the last two time points, should be interpreted with caution.

## Discussion

CIPN was a long-term problem for many patients in the SCOT trial regardless of the chemotherapy duration and regimen. We found a link between baseline neuropathy and long-term CIPN for up to 24 months after the start of treatment (we ceased statistical analysis at 24 months). Age, sex, and BMI were not associated with CIPN. Shorter duration of chemotherapy (3 months versus 6 months as already demonstrated by the SCOT trial) and CAPOX regimen were associated with lower severity of acute and long-term CIPN. We also observed the phenomenon of coasting. This is when symptoms worsen after treatment discontinuation.^5^^,^^34^ In general, CIPN symptoms improve after discontinuation of chemotherapy, and we observed this in both treatment arms. However, in patients treated for 3 months, we observed two peaks where CIPN worsened for the second time. Although we did not observe this in the 6-month arm, this could be due to the reducing frequency of data collection. After 6 months, the frequency of data collection was reduced from monthly to 3-monthly. Therefore, the two clear peaks and troughs could have been lost for the 6-month arm due to the less granular data. This could explain the difference in the CIPN patterns between the two trial arms. Our findings highlight key treatment-related (chemotherapy regimen) and patient-related (baseline neuropathy) factors associated with the increased severity of CIPN.

The relationship of acute CIPN with long-term CIPN has been shown in other studies.^34^^,^^35^ The significant relationship of baseline neuropathy with CIPN, as shown in this study, is less researched but equally important. A study by Wang et al. (2016) identified pre-chemotherapy touch sensation deficits as predicting acute CIPN.^36^ Sensory deficits before chemotherapy were also associated with CIPN at 6 and 12 months.^37^, ^38^, ^39^ Beyond that, few clinical trials of adjuvant oxaliplatin for colorectal cancer collected or investigated baseline neuropathy scores.^14^ The links of pre-chemotherapy neuropathy and acute CIPN are important for clinicians to help them to identify patients at greater risk of long-term CIPN. FACT/GOG-NTX-4 is a simple assessment of four items measured on a Likert scale, so it is easy to apply in clinical practice. This could aid decisions in early stages of chemotherapy and is important because dose modifications remain the only strategy for managing CIPN.^6^^,^^7^ Pharmacological and non-pharmacological interventions have largely failed, with the exception of duloxetine as the only recommended treatment for CIPN-related pian.^7^^,^^40^^,^^41^ In addition, cooling interventions such as cryotherapy show promise in preventing and treating CIPN, but more evidence is needed.^6^^,^^7^^,^^42^, ^43^, ^44^ A randomised controlled trial (RCT) studying cryotherapy in oxaliplatin showed a third of patients drop out due to discomfort.^44^

The literature remains conflicting on patient factors that predispose CIPN. A systematic review on risk factors for oxaliplatin-indicted neurotoxicity in patients with colorectal cancer found 15 studies reporting demographics, comorbidity (diabetes), and severity of acute neuropathy influencing CIPN.^9^ Out of the 15 studies, only 3 were prospective designs and in most of them the distinction between the acute and long-term CIPN was unclear or missing. Some other issues influencing the quality of the evidence in these studies pointed to small sample sizes and a lack of long-term follow-up data.^9^ Older age was reported as a risk factor in observational studies.^16^^,^^17^ However, a prospective study by Wong et al. (2019) have found age not to be associated.^45^ Similarly, in this study, we found no association of CIPN with age. Obesity was previously shown to be a significant predictor of CIPN severity.^46^^,^^47^ However, in our study we did not find BMI-related differences in CIPN.

Differences in CIPN occurrence and severity are clearly influenced by the cumulative dose of oxaliplatin. This was shown in a number of RCTs comparing 3 months to 6 months of treatment,^27^, ^28^, ^29^, ^30^ and a pooled analysis by the International Duration Evaluation of Adjuvant chemotherapy (IDEA) collaboration that established similar efficacy for stage III colon cancer.^25^ We enriched these findings by new knowledge because we showed that FOLFOX resulted in more severe CIPN than CAPOX, both acute and long-term. Despite similar cumulative doses between the two regimens, patients receiving CAPOX had significantly lower CIPN than those receiving FOLFOX in both SCOT arms. A large RCT by Yoshino et al. (2019) called ACHIEVE, studying 3 months versus 6 months oxaliplatin-based adjuvant chemotherapy for colon cancer, described similar findings of lower CIPN scores for patients receiving CAPOX than FOLFOX at 3 years.^30^ Soveri et al. (2019) concluded no difference between CAPOX and FOLFOX; however, it was a small study of only 144 patients.^35^ These findings need more research, but the potential hypothesis could be that in the CAPOX regimen patients had more time to recover between cycles than in the FOLFOX regimen (four cycles of CAPOX were delivered every 3 weeks and six cycles of FOLFOX were delivered every 2 weeks). Although the cumulative dose between the two regimens was similar, the different individual doses of oxaliplatin in the two chemotherapy schedules could have also influenced the observed differences. Dose delays and time differences between the cycles have been suggested as potential reasons for variability in CIPN.^4^^,^^24^^,^^48^ Further research of dose scheduling is important in relation to CIPN and our study provides evidence of the benefits of CAPOX over FOLFOX for those at higher risk of CIPN.

### Strengths and limitations

This study has some limitations; the FACT/GOG-NTX-4 is a shortened questionnaire that provides basic information about peripheral neuropathy occurrence and severity and does not provide both sensory and motor information as in the full 13-item FACT/GOG-NTX-13 questionnaire. This meant we were unable to define the differing characteristics of CIPN symptoms over time. Moreover, not all patients returned questionnaires and the amount of missing data increased with the duration of the follow-up. This limited statistical analysis that could be undertaken.

### Conclusions

In conclusion, CIPN is an important side-effect of oxaliplatin-containing chemotherapy that continues to be a problem in clinical practice despite dose reductions.^2^^,^^49^^,^^50^ Recommended guidance to prevent CIPN progression is to alter neurotoxic drug treatment by delaying, decreasing, or discontinuing it.^6^^,^^7^ However, we show that patients with prior neuropathy are more at risk of developing CIPN, and current guidelines do not adequately consider patient-related risk factors that should also be taken into account. Assessing baseline neuropathy with a simple neuropathy score could aid clinical decision making aimed at reducing long-term CIPN. More research is needed to stratify patients according to individual risks before chemotherapy, taking into account treatment factors. Shorter duration of chemotherapy is as efficacious as longer treatment in patients with colorectal cancer, and it has been shown to reduce CIPN.^25^, ^26^, ^27^, ^28^, ^29^, ^30^ In addition, our findings support the emerging evidence on the benefits of the CAPOX regimen through decreased CIPN. Patients with colorectal cancer form one of the largest groups of cancer survivors and are highly affected by CIPN.^51^ Therefore, an improved understanding of risk factors is an important survivorship priority for developing preventative and treatment strategies.

## References

[1] Han C.J., Yang G.S., Syrjala K. (2020). Symptom experiences in colorectal cancer survivors after cancer treatments: a systematic review and meta-analysis. Cancer Nurs.

[2] Bonhof C.S., van de Poll-Franse L.V., Wasowicz D.K. (2021). The course of peripheral neuropathy and its association with health-related quality of life among colorectal cancer patients. J Cancer Surviv.

[3] Park S.B. (2018). Chemotherapy-induced peripheral neuropathy: highlighting unmet needs. J Neurol Neurosurg Psychiatry.

[4] Beijers A.J., Mols F., Vreugdenhil G. (2014). A systematic review on chronic oxaliplatin-induced peripheral neuropathy and the relation with oxaliplatin administration. Support Care Cancer.

[5] Hertz D.L., Childs D.S., Park S.B. (2021). Patient-centric decision framework for treatment alterations in patients with chemotherapy-induced peripheral neuropathy (CIPN). Cancer Treat Rev.

[6] Jordan B., Margulies A., Cardoso F. (2020). Systemic anticancer therapy-induced peripheral and central neurotoxicity: ESMO-EONS-EANO Clinical Practice Guidelines for diagnosis, prevention, treatment and follow-up. Ann Oncol.

[7] Loprinzi C.L., Lacchetti C., Bleeker J. (2020). Prevention and management of chemotherapy-induced peripheral neuropathy in survivors of adult cancers: ASCO guideline update. J Clin Oncol.

[8] Staff N.P., Cavaletti G., Islam B. (2019). Platinum-induced peripheral neurotoxicity: from pathogenesis to treatment. J Peripher Nerv Syst.

[9] Pulvers J.N., Marx G. (2017). Factors associated with the development and severity of oxaliplatin-induced peripheral neuropathy: a systematic review. Asia Pac J Clin Oncol.

[10] Zajaczkowska R., Kocot-Kepska M., Leppert W. (2019). Mechanisms of chemotherapy-induced peripheral neuropathy. Int J Mol Sci.

[11] Kanat O., Ertas H., Caner B. (2017). Platinum-induced neurotoxicity: a review of possible mechanisms. World J Clin Oncol.

[12] Cavaletti G., Marmiroli P. (2020). Management of oxaliplatin-induced peripheral sensory neuropathy. Cancers (Basel).

[13] McCrary J.M., Goldstein D., Trinh T. (2019). Balance deficits and functional disability in cancer survivors exposed to neurotoxic cancer treatments. J Natl Compr Cancer Netw.

[14] Teng C., Cohen J., Egger S. (2022). Systematic review of long-term chemotherapy-induced peripheral neuropathy (CIPN) following adjuvant oxaliplatin for colorectal cancer. Support Care Cancer.

[15] Sałat K. (2020). Chemotherapy-induced peripheral neuropathy-part 2: focus on the prevention of oxaliplatin-induced neurotoxicity. Pharmacol Rep.

[16] Raphael M.J., Fischer H.D., Fung K. (2017). Neurotoxicity outcomes in a population-based cohort of elderly patients treated with adjuvant oxaliplatin for colorectal cancer. Clin Colorectal Cancer.

[17] Mizrahi D., Park S.B., Li T. (2021). Hemoglobin, body mass index, and age as risk factors for paclitaxel- and oxaliplatin-induced peripheral neuropathy. JAMA Netw Open.

[18] Miaskowski C., Mastick J., Paul S.M. (2017). Chemotherapy-induced neuropathy in cancer survivors. J Pain Symptom Manag.

[19] Ben Mahmoud I.T., Ben Said A., Berguiga S. (2023). Incidence and risk factors associated with development of oxalipatin-induced acute peripheral neuropathy in colorectal cancer patients. J Oncol Pharm Pract.

[20] Hershman D.L., Till C., Wright J.D. (2016). Comorbidities and risk of chemotherapy-induced peripheral neuropathy among participants 65 years or older in Southwest Oncology Group Clinical Trials. J Clin Oncol.

[21] Ottaiano A., Nappi A., Tafuto S. (2016). Diabetes and body mass index are associated with neuropathy and prognosis in colon cancer patients treated with capecitabine and oxaliplatin adjuvant chemotherapy. Oncology.

[22] Molassiotis A., Cheng H.L., Leung K.T. (2019). Risk factors for chemotherapy-induced peripheral neuropathy in patients receiving taxane- and platinum-based chemotherapy. Brain Behav.

[23] Kerckhove N., Collin A., Conde S. (2017). Long-term effects, pathophysiological mechanisms, and risk factors of chemotherapy-induced peripheral neuropathies: a comprehensive literature review. Front Pharmacol.

[24] Ewertz M., Qvortrup C., Eckhoff L. (2015). Chemotherapy-induced peripheral neuropathy in patients treated with taxanes and platinum derivatives. Acta Oncol.

[25] Andre T., Meyerhardt J., Iveson T. (2020). Effect of duration of adjuvant chemotherapy for patients with stage III colon cancer (IDEA collaboration): final results from a prospective, pooled analysis of six randomised, phase 3 trials. Lancet Oncol.

[26] Grothey A., Sobrero A.F., Shields A.F. (2018). Duration of adjuvant chemotherapy for stage III colon cancer. N Engl J Med.

[27] Iveson T.J., Kerr R.S., Saunders M.P. (2018). 3 versus 6 months of adjuvant oxaliplatin-fluoropyrimidine combination therapy for colorectal cancer (SCOT): an international, randomised, phase 3, non-inferiority trial. Lancet Oncol.

[28] Souglakos J., Boukovinas I., Kakolyris S. (2019). Three- versus six-month adjuvant FOLFOX or CAPOX for high-risk stage II and stage III colon cancer patients: the efficacy results of Hellenic Oncology Research Group (HORG) participation to the International Duration Evaluation of Adjuvant Chemotherapy (IDEA) project. Ann Oncol.

[29] Petrelli F., Rulli E., Labianca R. (2021). Overall survival with 3 or 6 months of adjuvant chemotherapy in Italian TOSCA phase 3 randomised trial. Ann Oncol.

[30] Yoshino T., Yamanaka T., Oki E. (2019). Efficacy and long-term peripheral sensory neuropathy of 3 vs 6 months of oxaliplatin-based adjuvant chemotherapy for colon cancer: the ACHIEVE phase 3 randomized clinical trial. JAMA Oncol.

[31] Iveson T., Hanna C., Iveson P. (2021). The early impact of the IDEA collaboration results: how the results changed prescribing practice. JNCI Cancer Spectrum.

[32] Mols F., Beijers T., Lemmens V. (2013). Chemotherapy-induced neuropathy and its association with quality of life among 2- to 11-year colorectal cancer survivors: results from the population-based PROFILES registry. J Clin Oncol.

[33] Cheng H.L., Lopez V., Lam S.C. (2020). Psychometric testing of the Functional Assessment of Cancer Therapy/Gynecologic Oncology Group—Neurotoxicity (FACT/GOG-Ntx) subscale in a longitudinal study of cancer patients treated with chemotherapy. Health Qual Life Outcomes.

[34] Pachman D.R., Qin R., Seisler D. (2016). Comparison of oxaliplatin and paclitaxel-induced neuropathy (Alliance A151505). Support Care Cancer.

[35] Soveri L.M., Lamminmäki A., Hänninen U.A. (2019). Long-term neuropathy and quality of life in colorectal cancer patients treated with oxaliplatin containing adjuvant chemotherapy. Acta Oncol.

[36] Wang X.S., Shi Q., Dougherty P.M. (2016). Prechemotherapy touch sensation deficits predict oxaliplatin-induced neuropathy in patients with colorectal cancer. Oncology.

[37] de Carvalho Barbosa M., Kosturakis A.K., Eng C. (2014). A quantitative sensory analysis of peripheral neuropathy in colorectal cancer and its exacerbation by oxaliplatin chemotherapy. Cancer Res.

[38] Reddy S.M., Vergo M.T., Paice J.A. (2016). Quantitative sensory testing at baseline and during cycle 1 oxaliplatin infusion detects subclinical peripheral neuropathy and predicts clinically overt chronic neuropathy in gastrointestinal malignancies. Clin Colorectal Cancer.

[39] Boyette-Davis J.A., Eng C., Wang X.S. (2012). Subclinical peripheral neuropathy is a common finding in colorectal cancer patients prior to chemotherapy. Clin Cancer Res.

[40] Meng J., Zhang Q., Yang C. (2019). Duloxetine, a balanced serotonin-norepinephrine reuptake inhibitor, improves painful chemotherapy-induced peripheral neuropathy by inhibiting activation of p38 MAPK and NF-κB. Front Pharmacol.

[41] Kim W., Chung Y., Choi S. (2017). Duloxetine protects against oxaliplatin-induced neuropathic pain and spinal neuron hyperexcitability in rodents. Int J Mol Sci.

[42] Jia J., Guo Y., Sundar R. (2021). Cryotherapy for prevention of taxane-induced peripheral neuropathy: a meta-analysis. Front Oncol.

[43] Coolbrandt A., Tobback H., Govaerts R. (2023). A randomized controlled trial of hand/foot-cooling by hilotherapy to prevent oxaliplatin-related peripheral neuropathy in patients with malignancies of the digestive system. ESMO Open.

[44] Beijers A.J.M., Bonhof C.S., Mols F. (2020). Multicenter randomized controlled trial to evaluate the efficacy and tolerability of frozen gloves for the prevention of chemotherapy-induced peripheral neuropathy. Ann Oncol.

[45] Wong M.L., Cooper B.A., Paul S.M. (2019). Age-related differences in patient-reported and objective measures of chemotherapy-induced peripheral neuropathy among cancer survivors. Support Care Cancer.

[46] Lee S., Ma C., Shi Q. (2022). Potential mediators of oxaliplatin-induced peripheral neuropathy from adjuvant therapy in stage III colon cancer: findings from CALGB (Alliance)/SWOG 80702. J Clin Oncol.

[47] Cox-Martin E., Trahan L.H., Cox M.G. (2017). Disease burden and pain in obese cancer patients with chemotherapy-induced peripheral neuropathy. Support Care Cancer.

[48] Beijers A.J., Mols F., Tjan-Heijnen V.C. (2015). Peripheral neuropathy in colorectal cancer survivors: the influence of oxaliplatin administration. Results from the population-based PROFILES registry. Acta Oncol.

[49] Lu L.C., Tsay S.L., Chang S.Y. (2019). Daily activity, mood, and quality of life in colorectal cancer patients with chemotherapy-induced peripheral neuropathy: a mediation effect analysis. Cancer Med.

[50] Mols F., Beijers A.J., Vreugdenhil G. (2015). Chemotherapy-induced peripheral neuropathy, physical activity and health-related quality of life among colorectal cancer survivors from the PROFILES registry. J Cancer Surviv.

[51] Sung H., Ferlay J., Siegel R.L. (2021). Global cancer statistics 2020: GLOBOCAN estimates of incidence and mortality worldwide for 36 cancers in 185 countries. CA Cancer J Clin.

